# Level and Factors Related to Unintended Pregnancy with a Brief Review of New Population Policies in Iran

**Published:** 2017-07

**Authors:** Khadijeh ASADI SARVESTANI, Aliyar AHMADI, Halimeh ENAYAT, Majid MOVAHED

**Affiliations:** Dept. of Sociology and Social Planning, Shiraz University, Shiraz, Iran

**Keywords:** Unwanted pregnancy, Contraceptive usage, Emergency contraception, Iran

## Abstract

**Background:**

High rate of unintended pregnancies in Iran is one of problems in family planning. The main goal of this study was to determine the rate of unintended pregnancies and to examine factors among married women in Shiraz City, Iran. It also discusses the possible effects of new population policies on the rate of unintended pregnancy.

**Methods:**

In this quantitative and survey study, data were collected by researcher-made questionnaire with interviewer from 400 pregnant women in Shiraz City referred to public and private health centers for prenatal care in 2013. Data were analyzed by SPSS both descriptively and analytically.

**Results:**

Overall, 17% of total pregnancies were unintended. The highest rate occurred among couples whose level of education was under diploma. In addition, women above 39 yr old experienced a higher rate of unintended pregnancy. The most popular methods were pills, withdrawal, and condom. The highest rate of unintended pregnancy was related to withdrawal. Knowledge about modern contraceptives particularly emergency contraceptives was low. Age, residence place, use of traditional contraception methods, knowledge about contraceptives, fear of side effects and couple agreement on contraception method were the main predictors of unintended pregnancy.

**Conclusion:**

There is still unmet need in family planning. The main predictors of unintended pregnancies are high prevalence of traditional contraception methods and insufficient knowledge about modern contraception methods. Policymakers should pay more attention to these issues. Furthermore, although Iranian policy makers are worried about low fertility, they need to be aware that new population policy through restriction of access to family planning services is effective, but also may exacerbate the problem by leading to a higher chance of unintended pregnancy.

## Introduction

Iran’s advances in family planning program lead to a dramatic decline in total fertility rate (TFR). In more details, fertility rate dropped from 6.5 in 1986 to 1.6 in 2012 (1). Furthermore, Iran has the highest rate of contraceptive usage among Muslim countries, and this rate is comparable with advanced countries such as France and Germany (2). However, there are some unsolved problems pertaining to family planning such as unmet need and unintended pregnancy. Obviously, women’s mental and physical health, as well as their mortality rate in reproductive ages, is very dependent on their reproductive behaviors, childbirths, pregnancies, and those complications (3).

Unintended pregnancy is a potential hazard for a woman’s life in productive ages. Statistically, 40% of 213 million pregnancies that occurred worldwide in 2012 were unintended, about the same proportion as in 2008, when 42% of all pregnancies globally were unintended (4). “An unintended pregnancy is a pregnancy either mistimed (i.e., they occur earlier than desired) or unwanted (i.e. they occur when no children or no more children are desired) at the time of conception” (5). Due to socioeconomic development, modernization, and urbanization, couples desire to have fewer children, as a result, they spend more time to avoid from unintended pregnancies (4).

An unintended pregnancy is a global challenge, and it is a main cause of abortion worldwide, particularly in the developing world (6). Unintended pregnancies are associated with an array of negative health, economic, social, and psychological outcomes for women and children, their families, society and nation (7, 8). Consequently, an integral part of the UN Millennium Development Goals is preventing unintended pregnancies (9).

In the case of Iran, despite the great success of Iran in family planning programs, about 35% of pregnancies were unintended. However, about 42% of women with unwanted pregnancy were practicing a contraception method (10). A large proportion of women in reproductive age are at the risk of unwanted pregnancy and subsequently induced and hidden illegal abortion (11). Today, there are many modern methods of contraception, but the important question is that why is the rate of unintended pregnancy relatively high?

The effectiveness of birth control methods vary, and that method failure is a contributing factor to unwanted pregnancies, but the effectiveness of most methods depends strongly on how conscientious a user is in its use (user failure). There is a distance between theoretical efficacy and effectiveness in practice (12). For example, OC (oral contraceptive) is the most popular modern contraception method in Iran. Statistically, OCs have first-year pregnancy rates of less than 1% pregnancy rates if used perfectly, but findings of the most recent Iran Demographic and Health Survey (IDHS) indicates that only 51.5% of women taking OCs used them correctly and about 6% of the unintended pregnancies among married women occurred among OC users (13). Accordingly, understanding factors leading to method failure and subsequently unintended pregnancy is of great importance.

Moreover, although successful in family planning, Iran has changed its approach to FP policies in recent years. Iranian policymakers are worried about low fertility rate; they are planning to improve it. For example, Iran’s parliament has recently banned vasectomy and tubectomy except to save a person’s life (1). Accordingly, with attention to some changes in population policies from pronatalist to anti-pronatalist, the question arises that how these changes can affect the rate of unintended pregnancy.

The main goal of this study was to determine the rate of unintended pregnancies and to examine factors among married women in Shiraz City-Iran. It also discusses the possible effects of new population policies on the rate of unintended pregnancy.

## Materials and Methods

The method of this study was quantitative and its technique was surveyed. This study was conducted on pregnant women referred to public and private health centers of Shiraz, Iran for prenatal care. The data was collected by researcher-made questionnaire with interviewer from 400 randomly selected pregnant women in Shiraz City who referred to public and private health centers for prenatal care. In more details, 200 questionnaires collected from pregnant women referred to public health centers and 200 questionnaires collected from pregnant women referred to private health centers. The questionnaire was consist of questions regarding place of residence, marital age, educational level, working status, level of knowledge about contraceptives and their side effects, last contraceptive method used prior to the recent pregnancy, and intention for abortion.

Social science and health professionals determined validity of the questionnaire. Furthermore, the reliability of the questionnaire was determined by doing a pilot study and Cronbach’s alpha test with (α = 0.75). The data was analyzed by the SPSS statistical software ver. 20 (Chicago, IL, USA).

## Results

Seventeen percent of pregnancies are unintended. The highest rate of unintended pregnancy is related to women above 35 yr and to homemakers (Table 1).

**Table 1:** Rate of unwanted pregnancy by social and demographic and accessibility factors (n=68)

**Variable**	**Frequency**	**Percentage**
**Age groups (yr)**		
15–19	12	17.2
20–24	10	14.9
25–29	7	10.7
30–34	18	27
35^+^	22	30.2
**Job**		
Housewife	63	92.6
Employed	5	7.4
**Education Level**		
Diploma and Under Diploma	56	82.4
Above Diploma	12	17.6
**Education level of Husband**		
Under Diploma and Diploma	58	85.3
Above Diploma	10	14.7
**Agreement with husband about contraception method**		
Yes	30	44.1
No	38	55.9
**Place of receiving contraceptive**		
Governmental centers	33	48.5
Non-Governmental centers	29	42.6
Unknown	6	8.8
**Satisfaction of information received**		
Yes	30	44.71
No	38	55.29

In terms of education level, most unintended pregnancies occurred among couples with education level of diploma and under diploma. About 56% of women with unintended pregnancy did not have agreement with their husband about contraception method. Finally, 48.5% of women received contraception device or consultation from governmental health center and about 42.6% from non-governmental centers. However, half of women believed that personnel of health centers (governmental and non-governmental) did not give enough information about contraceptives.

**Fig. 1:**
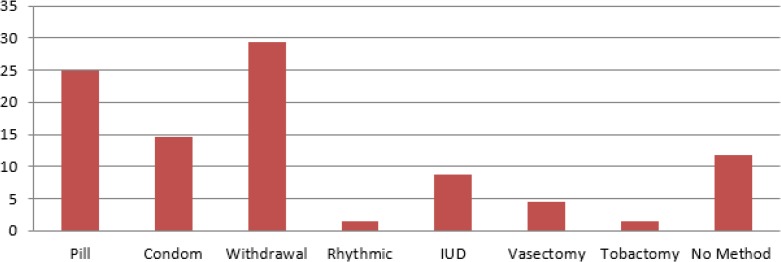
Kind of contraception method before pregnancy among women with unintended pregnancy

**Fig. 2:**
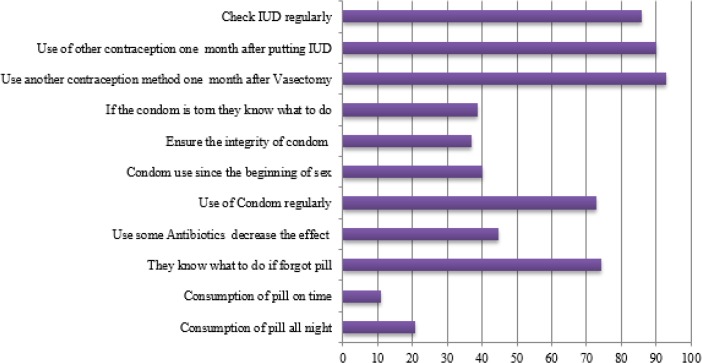
Women knowledge about some contraception methods

Fig. 1 illustrates percentage distribution of women according to contraception method. Fig. 2 shows women’s knowledge about contraception method. The highest rate of unintended pregnancies is related to women whose contraception method was withdrawal as a traditional method. The second popular method among women was pill.

In more details, about 25% of women have become pregnant while using the birth control pill. About 20% of pill users did not use it daily and about 10% of them did not use it on time. Thirty percent of women said that they did not know what to do in case they forgot to take pill. In addition, more than 40% of pill users did not know that some antibiotics could decline the effectiveness of pills.

After the pill near to 14% of women acknowledged that condom was their method of contraception. However, less than 40% of condom users check the condom in terms of integrity. More importantly, approximately 60% of them did not know what to do if condom was torn during the intercourse. In addition, 40% of condom users said that they did not use condom since the beginning of sex relationship and about 30% of them did not use condom regularly. Another male method is Vasectomy.

About 3% of women said that they became pregnant while their husbands did vasectomy. About 10% of them did not use another contraception method after one month of surgery. The third most popular modern contraception was IUD. Although it is necessary to check IUD regularly, about 15% of women acknowledged that they did not check it on a regular base. Moreover, only 10% of them have used another contraception method one month after putting IUD. Finally, more than 10% of women did not use any contraception method, which is a sign of unmet need (Fig. 1).

**Fig. 3:**
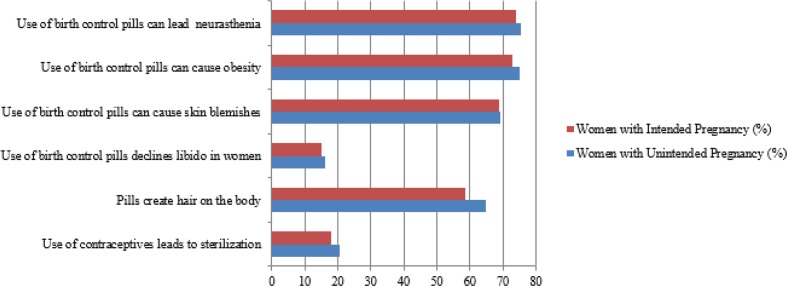
Fear of side effect of pill among women with unintended and wanted pregnancy

Fear of side effects of the contraceptive can affect contraceptive usage, and subsequently, the rate of unintended pregnancy. Fig. 3 indicates the fears of side effects of pill usage in terms of intention of pregnancy (whether wanted or unwanted). More than 60% of women with both unintended pregnancy and wanted pregnancy believed that pills could lead to neurasthenia, skin blemishes, obesity, and extra hair on body. The percentage of women with unintended pregnancy who believed in these aforementioned side effects is higher compared to women with intended pregnancy. However, results by Independent *t-*test showed that their difference is not significant.

Table 2 demonstrates the prediction of unintended pregnancy through some variables. Multiple logistic regression analysis was conducted to examine how selected independent variables were related to the likelihood of unintended pregnancy.

**Table 2:** Prediction of contraceptive usage

**Predictors**	**B**	**S.E.**	**Wald**	**df**	**Sig.**	**Exp (B)**
Respondent's Age in Year	.063	.020	10.04	1	.002	.939
Education in Year	−.157	.068	5.6	1	.021	.626
Knowledge about Contraception	−.211	.056	13.94	1	.000	1.23
Fear of Side effects	.044	.024	3.2	1	.000	.282
Couple Agreement on Contraception Method	−.869	. 319	7.4	1	.006	.419
Using Traditional Contraception Method	.275	.092	4.0	1	.044	.660
Living in Urban areas	−1.41	.377	13.9	1	.000	.245
Constant	−1.03	1.19	.69	1	.305	.339

Cox & Snell R2 =.148 / Nagelkerke R2 =.246 / Hosmer and Lemeshow Test; Sig=. 064

Results of Logistic Regression reveal that seven variables have significant relationship with unintended pregnancy. The age is one of predictors that have positive relationship with unintended pregnancy (B=0.063, Wald=10.04, *P*<0.05). In more detail, the chance of unintended pregnancy goes up with increase in age. Education is also predictor of unintended pregnancy (B= −0.157, Wald=5.6, *P*<0.05). In other words, chance of unintended pregnancy comes down with increase in education level.

The next predictor is couple agreement on contraception method (B= −0.980, Wald= 5.3, *P*<0.05). In more details, the possibility of unintended pregnancy among couples who do not agree on contraception method is 5.3 times more than couples that have an agreement.

The next predictor is knowledge about contraceptives (B= −0.211, Wald= 13.94, *P*<0.05). Fear of side effects of contraceptives is another predictor that positively influences the chance of unintended pregnancy (B=0.044, Wald= 13.94, *P*<0.05). The higher level of knowledge, the fewer the chances of unintended pregnancy. Using traditional contraction methods is another predictor of unintended pregnancy (B=0.275, Wald=3.2, *P*<0.05). In other words, chance of unintended pregnancy among women applying using traditional methods is four times more than women applying using modern contraceptives. Finally, place of residence is another predictor, as can be seen living in urban negatively influence the chance of unintended pregnancy (B= −1.03, Wald=0.69, *P*<.05). The chance of unintended pregnancy was lower among urban women compared to rural women.

## Discussion

According to the present study, the rate of unwanted pregnancy was 17.1% (14.7%). The highest prevalence of unwanted pregnancies was among women above 35 yr of age. Therefore, older women are more vulnerable while another study in Iran found that the risk of unwanted pregnancy among younger women is higher (14). Another influential factor was couples’ education level. The highest rate of unwanted pregnancy occurred among couples with education level of diploma and lower. Obviously, couples with lower education levels have lower knowledge about contraceptive, as a result, the rate of contraceptive usage among them is lower and method failure is higher. Furthermore, educated couples are more likely to discuss the timing and number of children and the use of different contraceptive methods (15). Accordingly, education level has a great effect on unintended pregnancy, it would be better for family planning programs to have more focus on the lower-educated group of people.

One of the factors that can affect by education is couple agreement on contraception method. As discussed earlier, more than 50% of couples did not agree on contraception method and according to regression results, this factor was one of predictors of unintended pregnancy. Similarly, wives with the perception that their husbands approved of contraceptive usage, were more likely to use contraceptives, and a modern method, than wives with the perception that their husbands disapproved (16, 17). Accordingly, men participation in family planning programs can increase a couple’s contraceptive. Clearly, couple’s agreement on contraception method is not affected only by education level, thus, understanding factors contributing to agreement of couples on contraception method and men involvement in family planning can help policymakers in tackling the problem of unintended pregnancy.

The most popular contraceptive methods among women with unwanted pregnancy were withdrawal, pill, and condom. This is similar to studies conducted in other cities of Iran such as Hamadan (18). In addition, withdrawal was the most used method among these women. Withdrawal method can be as effective as most non-hormonal barrier methods. Statistically, 4% of women using this method perfectly can expect an unintended pregnancy within the first year of use. However, user error can be very high using this method – the unintended pregnancy rate in the first year is 27% (19). Factors such as no need for a doctor’s order, low side effects, free procedure, no health concerns, lack of trust to modern methods, comfortable using of withdrawal method, and reluctance of their husbands to use other methods are the reasons for choosing this method (20).

Pill was the most popular modern method but most women did not use it correctly. Although the rate of using the pill among Iranian women is close to that of developed countries (21), the rate of unintended pregnancy among pill users is high because they do not use it truly.

In addition, a considerable percentage of women whose partners use condom said that they did use condom on a regular basis, did not check condom before using, did not have information about the emergency method when condom was torn during intercourse and did not use condom since the beginning of sex relationship. Generally, problems related to condom can be divided into two categories. First, although condom can play a vital role in decreasing the rate of unintended pregnancies and sexually transmitted infections, its using rate in Iran is low even in comparison to some Asian countries. For example, statistics demonstrate that the rate of using condom in Hong Kong SAR of China is 46% and in Japan 41% (21). Second, condom users do not have enough information about it.

Furthermore, although emergency contraception methods can play a vital role in preventing the considerable percentage of unwanted pregnancies, but women’s level of knowledge about emergency contraception is very low. In other words, the main obstacle in the emergency contraception methods (ECs) is not their side effects or failure. The problem is a low knowledge or negative attitude about ECs (22, 23). Finally, 12% of women under this study did not use any contraception methods while they had no plan for pregnancy, which is a sign of unmet need in family planning. Unmet need can result from supply side factors such as unavailable family planning service or other constraints that prevent individuals from acting on their fertility goals (24).

In spite of socioeconomic and cultural factors, one of the factors contributing to unintended pregnancy is access to family planning services. Centers offering family planning can play vital role in supporting women to achieve their fertility goals. The role of family planning centers is not limited to providing contraception devices but they can play an important role in providing knowledge about contraceptives according to needs of clients. In Shiraz City, there are many governmental and non-governmental centers providing family planning services but a considerable percentage of respondents with unintended pregnancy believed that the personnel of health centers did not give enough information about contraception methods.

Back to the discussion on changes in population policies, concerning problems such as the rate of unintended pregnancy, the rate of unmet need and the rate of method failure in a situation that family planning services were free and accessible; any limitation in providing family planning services can increase the prevalence of these problems. Obviously, population control policies are multifaceted policies, and various aspects of their implementation are of utmost importance. The role of contraception in decreasing fertility rate in Iran has been overestimated by policymakers. In fact, fertility rate in Iran began to decline since 1985, four years before the introduction of new FP policies in 1989. In addition, only 61% of the reduction in TFR could be attributed to FP practices (1, 11).

Furthermore, the experience of a country such as Romania is a good example of the fact that restricting access to family planning for increasing the fertility rate not only is ineffective but also may lead to many implications. In 1966, the Romanian Government dramatically changed its population policy concerning the low rate of population growth. It introduced a number of measures to increase the fertility rate that made abortion legally available only in limited circumstances, restricted access to contraception, and increased allowances for large families. Due to these efforts, birth rate had increased for a short time but it started to decrease once again in 1967 and reached the 1966 level (14.3 births per 1000) in 1983. In spite of government restrictions on abortion, the abortion ratio also started to increase in 1967. During 1965–1983 maternal mortality reached heights unprecedented in Europe, from 85 deaths per 100000 live births in 1965 to 170 in 1983. Statistically, illegal, unsafe abortion was the major cause of maternal mortality, accounting for more than 80% of maternal deaths between 1980 and 1989 (1, 25).

Although in the case of Iran, access to family planning services is not totally banned but this restriction can lead to many implications. In more details, about 50% of women receive their contraceptives from governmental health centers; as a result, any restriction in access to these services is equal to increasing problems such as unintended pregnancy, illegal abortion, and maternal mortality. Additionally, rates of traditional methods such as withdrawal are high and rates of modern methods such as condom and vasectomy are low. Consequently, restriction in access can lead to an increase in traditional contraception method and decline of modern contraceptives. These issues are more significant in terms of couples from low socioeconomic categories and rural couples. For this group, costs of contraceptives (such as visiting specialists and buying contraception devices) can lead to stopping using modem contraceptives. Consequently, with attention to the increasing population of women at childbearing ages, health of women and children do not sacrifice of new population policies.

## Conclusion

Family planning in Iran still has some problems. Accordingly, policy makers should consider these issues in employing of new population policy. Otherwise, any non-scientific program can increase the prevalence of current problems and may create new problems in family planning program.

## Ethical Considerations

Ethical issues (Including plagiarism, consent, misconduct, data fabrication and/or falsify-caution, double publication and/or submission, redundancy, etc.) have been completely observed by the authors.
